# Value of Assessing Peripheral Vascularization with Micro-Flow Imaging, Resistive Index and Absent Hilum Sign as Predictor for Malignancy in Lymph Nodes in Head and Neck Squamous Cell Carcinoma

**DOI:** 10.3390/cancers13205071

**Published:** 2021-10-10

**Authors:** Petra K. de Koekkoek-Doll, Sander Roberti, Michiel W. van den Brekel, Monique Maas, Laura Smit, Regina Beets-Tan, Jonas Castelijns

**Affiliations:** 1 Department of Radiology, The Netherlands Cancer Institute, 1066 CX Amsterdam, The Netherlands; m.maas@nki.nl (M.M.); r.beetstan@nki.nl (R.B.-T.); j.castelijns@nki.nl (J.C.); 2 Department of Epidemiology and Biostatistics, The Netherlands Cancer Institute, 1066 CX Amsterdam, The Netherlands; s.roberti@nki.nl; 3 Department of Head and Neck Surgery and Oncology, The Netherlands Cancer Institute, 1066 CX Amsterdam, The Netherlands; m.vd.brekel@nki.nl; 4 Department of Maxillofacial Surgery, Amsterdam University Medical Center, University of Amsterdam, 1105 AZ Amsterdam, The Netherlands; 5 Department of Pathology, The Netherlands Cancer Institute, 1066 CX Amsterdam, The Netherlands; l.smit@nki.nl

**Keywords:** SCC, head and neck, lymph nodes, ultrasound, micro-flow imaging, hilum sign, resistive index

## Abstract

**Simple Summary:**

Ultrasound-guided fine needle aspiration cytology (USgFNAC) is commonly used for N-staging in head and neck squamous cell carcinoma (HNSCC). The specificity of USgFNAC is always in the order of 100% as false positive cytology is rare. The difference in sensitivity is mainly attributable to selection of the lymph nodes to aspirate and aspiration technique. The aim of this study was to improve the selection criteria of lymph nodes to aspirate. Ultrasound features of nodes such as a short axis diameter, S/L ratio, loss of a fatty hilum sign, resistive index, and peripheral or mixed hilar and peripheral vascularization, obtained by Micro-flow imaging (MFI), which is a new technique to obtain micro-vascularization, were evaluated. To calculate the sensitivity and PPV of each feature, data of sonographic findings and cytological results of all aspirated nodes were statistically analyzed. We found that next to size, peripheral vascularisation obtained by MFI and absent hilum sign have a high predictive value for malignancy and should be added as selection criteria for fine needle aspiration in lymph nodes.

**Abstract:**

Ultrasound-guided fine needle aspiration cytology (USgFNAC) is commonly used for nodal staging in head and neck squamous cell cancer (HNSCC). Peripheral vascularity is a described feature for node metastasis. Micro-flow imaging (MFI) is a new sensitive technique to evaluate micro-vascularization. Our goal is to assess the additional value of MFI to detect malignancy in lymph nodes. A total of 102 patients with HNSCC were included prospectively. USgFNAC was performed with the Philips eL18–4 transducer. Cytological results served as a reference standard to evaluate the prediction of cytological malignancy depending on ultrasound features such as resistive index (RI), absence of fatty hilum sign, and peripheral vascularization. Results were obtained for all US examinations and for the subgroup of clinically node-negative neck (cN0). USgFNAC was performed in 211 nodes. Peripheral vascularization had a positive predictive value (PPV) of 83% (cN0: 50%) and the absence of a fatty hilum had a PPV of 82% (cN0 50%) The combination of peripheral vascularization and absent fatty hilum had a PPV of 94% (cN0: 72%). RI (threshold: 0.705) had a PPV of 61% (cN0: RI-threshold 0.615, PPV 20%), whereas the PPV of short axis diameter (threshold of 6.5mm) was 59% for all patients and 19% in cN0 necks (threshold of 4 mm). Peripheral vascularization assessed by MFI and absent hilum has a high predictive value for cytological malignancy in neck metastases. Next to size, both features should be used as additional selection criteria for USgFNAC.

## 1. Introduction

One of the most important predictors for the survival of patients with head and neck squamous cell carcinoma (HNSCC) is the nodal status [1]. Metastatic disease that spreads from the primary lymph node to distant organs causes 90% of all HNSCC deaths. Accurate staging is therefore essential for prognostication and optimal treatment planning with the goal to obtain the best cure and avoid treatment morbidity [2,3].

Neck palpation for lymph nodes in patients with HNSSC has a sensitivity and specificity to detect metastatic disease of 60–70% [4]. That means that around 30 to 40% of the nodal metastases are clinically occult (cN0).

Commonly used imaging tools to detect these occult metastases are ultrasound (US), magnetic resonance imaging (MRI), contrast enhanced computer tomography (CT), FDG PET-CT, and ultrasound-guided fine needle aspiration-cytology (USgFNAC). MRI and CT are frequently used to stage the primary tumor and neck, but use morphological criteria for metastases with a relatively low accuracy (74–78%) [5]. 18FDG PET-CT enables, next to the morphological criteria, use of metabolic criteria, and is reported to be superior to MRI and CT with a sensitivity and specificity of 84% and 96%, respectively [6]. However, for cN0 neck, with only small metastases, the sensitivity is in the range of 40–60% and thus not very high [7].

USgFNAC can reduce the risk from an initial risk of occult metastases of 40% to a risk of 10–20%, which can be considered acceptable to refrain from elective treatment, although this remains a controversial topic [8].

High-resolution US to guide FNAC is an important diagnostic tool and well established. Gray scale ultrasound enables assessment of morphological criteria such as nodal size, nodal boundary, cystic transformation, or other internal reflective patterns, fatty hilum sign, surrounding edema, or infiltration of the surrounding tissue [9,10,11].

Power Doppler sonography has been shown to be a reliable method for the assessment of the vascularity of cervical lymph nodes [12] It allows to evaluate the pattern of the intra-nodular macro vascularization and to measure the resistive index (RI). It has been shown that normal lymph nodes have a hilar vascularity while metastatic nodes may have a peripheral or mixed hilar and peripheral vascularity [13,14]. The RI is reported to be higher in metastatic nodes than in reactive lymph nodes. In a recent review, Ying et al. described an optimal cut-off for RI at 0.7 for differentiating between metastatic and reactive lymph nodes, with a sensitivity of 47–81% and a specificity of 81–100% [15]. Because Doppler ultrasound techniques display the changes of macro vascularization, vascularity is often not detected in small lymph nodes [16].

Micro-flow imaging (MFI) is a relatively new mode designed to detect small vessel flow with high resolution and minimal artefacts. Recent studies have shown that MFI has a higher sensitivity to detect tumoral vascularity compared with color Doppler imaging (CDI) and power Doppler imaging (PDI) [17,18,19]. MFI can also improve the visualization of peripheral vascularization in neck lymph nodes as a feature of metastasis. To our knowledge the value of MFI has not been examined in cervical lymph nodes.

The aim of this study was to evaluate the additional value of peripheral vascularization in lymph nodes as assessed by MFI as a criterion to diagnose metastasis or select lymph nodes to be punctured by USgFNAC next to other criteria such as nodal size, fatty hilum sign, and RI obtained in the same nodes.

## 2. Materials and Methods

### 2.1. Patients

A total of 102 patients with histopathologically proven HNSCC were included prospectively; data were analyzed retrospectively. All patients were referred for nodal staging (N-staging) by USgFNAC. USgFNAC was performed in all suspicious nodes as in a usual clinically setting. The median age was 65 years (range: 34–87yrs); 27/102 (26%) patients were female, and 73/102 (72%) patients were male (Table 1).

### 2.2. Ultrasound and USgFNAC

Ultrasound was performed with an EpiQ7 ultrasound system (Philips, Bothell, WA, USA), using a dedicated protocol for N-staging of HNSCC. The eL18–4 transducer (Philips) was used for conventional ultrasound (B mode), color Doppler sonography (CDI) for measurement of the resistive index (RI), and micro-flow imaging (MFI) for assessing peripheral vascularization. Before aspiration, the short axis diameter and morphological features of the node were assessed. MFI with monochrome subtraction mode imaging was used to detect the presence or absence of peripheral vascularity. The sampling window was placed such that it covered the whole lymph node and surrounding tissue. Images of the nodes with present or absent hilum sign and peripheral vascularization were obtained and categorized. The RI is calculated from the index of the peak systolic blood velocity (Vmax) relative to the minimal diastolic flow velocity (Vmin) reflecting the resistance of the microvascular flow distal of the measurement. All RI measurements were obtained in the hilus if present, and within the node otherwise. To avoid pulsation noise from the carotid artery while maximizing blood vessel visualization, MFI and color gain were adjusted dynamically.

**Figure 1:**
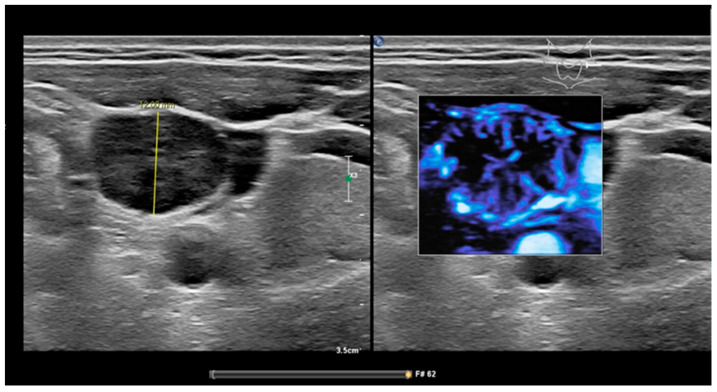
MFI of peripheral vascularity in a patient with oropharyngeal SCC. At cytology metastasis SCC, MFI shows a strong peripheral vascularity which indicates malignancy; fatty hilum sign is absent.

**Figure 2:**
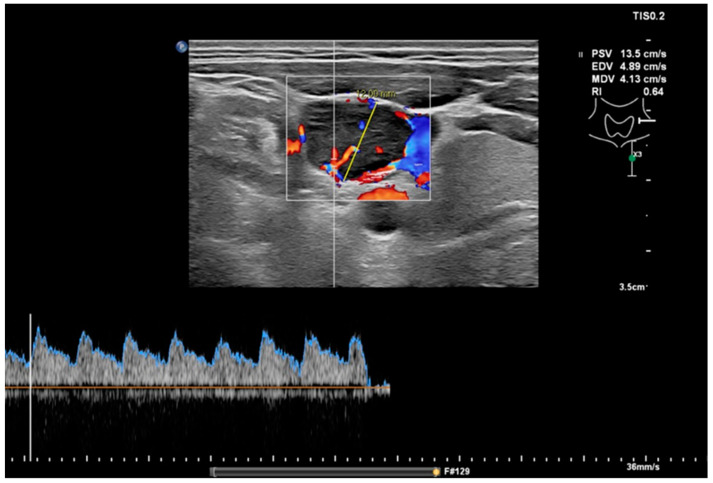
Measurement of the RI in the same node as in Figure 1 with a value of 0.64, which would indicate a benign node.

**Figure 3:**
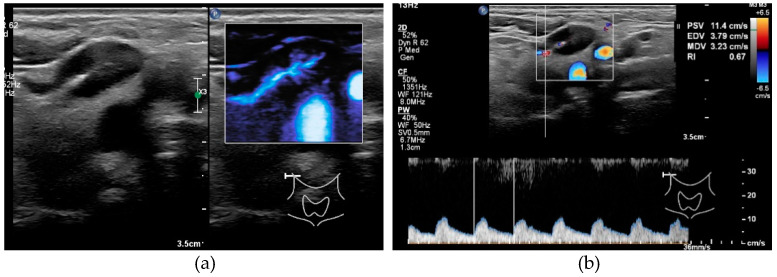
Ultrasound features of a benign node. (**a**) Hilum sign in a benign node, no peripheral vascularity. (**b**) Measurement RI 0.67.

USgFNAC was performed in all nodes with a short axis diameter ≥ 7 mm, or in nodes <7 mm with loss of a fatty hilum sign, peripheral or mixed hilar and peripheral vascularity, a round shape, or an asymmetric thickened cortex (Figure 1, Figure 2 and Figure 3).

In all nodes, USgFNAC was performed with a 21G needle and cytological results served as the reference standard in assessing the predictive value of the US features. All measurements and FNAs took place by the same experienced neuroradiologist with over 10 years’ experience in head and neck USgFNAC (P.K.d.K.-D).

### 2.3. Cytology

FNAC material was processed in smears, air dried, and stained with Giemsa stain. Part of the material was fixed in 10 mL 4% formalin and embedded in paraffin for further immunohistochemistry, if necessary, according to routine diagnostic workup. All samples were evaluated by experienced cytopathologists.

### 2.4. Statistical Analysis

Data of sonographic findings and cytological results of USgFNAC were statistically analyzed for all aspirated nodes and separately for two subsets of aspirated nodes: nodes from clinically node-negative necks (cN0) and nodes with a short axis diameter of 6 mm or less.

In contrast to most reports in the literature, we calculated sensitivity and other parameters per aspirated lymph node, not per neck side or patient, as we were interested in the optimal criteria and not the reliability in clinical practice. We assessed the performance of nodal size (short axis diameter and short/long axis(S/L) ratio, dichotomized using S/L > 0.5, absent fatty hilum sign, presence of peripheral vascularization and RI in predicting cytological malignancy of an aspirated lymph node, using sensitivity, specificity, positive predictive value (PPV) and negative predictive value (NPV). For binary (including dichotomized) variables, these metrics were determined using the 2 × 2 confusion matrix. For the continuous variables (short axis diameter and RI), a threshold was first determined using ROC curve analysis such that the sensitivity was at least as large as for the classification using peripheral vascularization obtained by MFI. For short axis diameter, an additional threshold based on the literature was used (6 mm for all nodes, and 4 mm for cN0 subgroups) [20]. Additionally, the smallest cutoff with a corresponding PPV of 100% in all nodes was determined for the short axis diameter.

All analyses with RI were done on the subset of lymph nodes with an available RI measurement. Measurement of the RI failed in 8% of the nodes, mainly in tiny or necrotic nodes. The performance of peripheral vascularization obtained by MFI was also assessed in two additional subsets of nodes: nodes with absent fatty hilum sign, and nodes from clinically node-negative neck with absent fatty hilum sign. Note that any PPV estimate obtained in these subset analyses is by definition the same as would be obtained from combining the features, e.g., the PPV for peripheral vascularization in nodes with absent fatty hilum is the same as the PPV that would be obtained in the set of all nodes by predicting malignancy for nodes with both absent fatty hilum sign and peripheral vascularization.

We assessed whether short axis diameter or S/L ratio differed significantly between cytologically malignant and cytologically benign nodes as shown by USgFNAC, within all nodes and in the subset cN0. Further, we assessed whether short axis diameter or short/long ratio of malignant nodes differed significantly between patients with cN+ and cN0 stage. For this, we used linear mixed effects models with short axis diameter or ratio as the dependent variable, the categorical variable of interest (cytological malignancy or cN stage) as a fixed effect, and patient number as a random intercept. The significance of the categorical variable was then determined using a likelihood ratio test with a 5% significance level.

To determine 95% confidence intervals for the obtained predictive performance measures, accounting for the dependence between nodes from the same patient, we used a bootstrap procedure with 10,000 iterations. During each iteration, a bootstrap sample was generated by resampling patients with a replacement from the original dataset. Then, the sensitivity, specificity, PPV, and NPV were obtained for all variables as described above. From the full set of these results, the 95% bias-corrected accelerated confidence interval [21] was determined. This was not possible for all metrics, as some metrics had the same value in all bootstrap samples. Further, some bootstrap samples did not have at least one malignant and benign node in each category for certain variables, resulting in a missing value for that metric. When for a certain metric the computation of the BCa interval was not possible, when at least 5.5% of bootstrap estimates were missing, or when the BCa interval used order statistics among the first or last 10, the 95% binomial proportion confidence interval was computed for that metric instead.

All analyses were performed with R statistical software, version 3.6.1 (R Core Team (2021). R: A language and environment for statistical computing. R Foundation for Statistical Computing, Vienna, Austria).

## 3. Results

### 3.1. Analysis in Entire Set of Nodes

USgFNAC was performed in 211 nodes from 102 patients. (Table 1) The mean number of USgFNAC punctures per patient was 2.07 (range 1–5). Out of 211 nodes, 8 (4%) were inconclusive at cytology, 95 (45%) proved to be malignant, and 108 (51%) did not show malignant cells. Nodes that were inconclusive at cytology were excluded from further analyses. 

#### 3.1.1. Short Axis Diameter

Malignant nodes at cytology had a significantly larger short axis diameter than benign nodes (*p*-value <0.0001). The mean short axis diameter of all nodes was 9.8 mm (SD 6.4), while it was 6.7 mm (SD 2.1) for cytologically benign nodes and 13.3 mm (SD 7.7) for cytologically malignant nodes. 

Predicting cytological malignancy for short axis diameters ≥ 6.5 mm had a sensitivity of 0.88 (95% CI 0.80–0.95), a specificity of 0.45 (95% CI 0.19–0.81), a PPV of 0.59 (95% CI 0.45–0.82), and an NPV of 0.82 (0.59–0.89; Table 2). With a threshold of 6.0 mm (based on the literature), the sensitivity was 0.95 (95% CI 0.89–0.98), the specificity was 0.25 (95% CI 0.17–0.35), the PPV was 0.53 (95% CI 0.43–0.62), and the NPV was 0.84 (95% CI 0.68–0.94; Table 2 and Table 3).

The lowest cut-off for the short axis diameter with a PPV of 100% was 14 mm.

#### 3.1.2. S/L Ratio

The mean S/L ratio was 0.62 (SD 0.17) for all nodes, 0.55 (SD 0.16) for cytologically benign nodes, and 0.71 (SD 0.15) for cytologically malignant nodes. Malignant nodes had a significantly larger S/L ratio than benign nodes (*p*-value <0.0001). Using S/L ratio to predict cytological malignancy for nodes with a ratio > 0.5 had a sensitivity of 0.88 (95% CI 0.82–0.93), a specificity of 0.45 (95% CI 0.37–0.53), a PPV of 0.59 (95% CI 0.49–0.67), and an NPV of 0.82 (95% 0.69–0.90; Table 2).

#### 3.1.3. Resistive Index

RI was successfully obtained for 187/203 (92%) nodes. Predicting cytological malignancy for nodes with RI ≥ 0.705 had a sensitivity of 0.88 (95% CI 0.78–0.93), a specificity of 0.54 (95% CI 0. 0.34–0.70), a PPV of 0.61 (95% CI 0.50–0.74), and an NPV of 0.85 (0.72–0.92; Table 2).

#### 3.1.4. Peripheral Vascularization 

Peripheral vascularization as shown by MFI was present in 100/203 (49.3%) nodes. Predicting cytological malignancy had a sensitivity of 0.87 (95% CI 0.80–0.93), a specificity of 0.84 (95% CI 0.77–0.90), a PPV of 0.83 (95% CI 0.74–0.90), and an NPV of 0.88 (0.80–0.94; Table 2 and Table 3).

#### 3.1.5. Absent Hilum Sign

Hilum sign was absent in 97/203 (47.8%) nodes and had a sensitivity of 0.84 (95% CI 0.77–0.90), a specificity of 0.84 (95% CI 0.77–0.90), a PPV of 0.82 (95% CI 0.72–0.90), and an NPV of 0.86 (0.76–0.92) in predicting cytological malignancy. 

Among nodes with absent fatty hilum sign, peripheral vascularization obtained by MFI predicted cytological malignancy with a sensitivity of 0.92 (95% CI 0.85–0.97), a specificity of 0.71 (95% CI 0.45–0.89), a PPV of 0.94 (95% CI 0.86–0.98), and an NPV of 0.67 (0.38–0.85; Table 2 and Table 3).

### 3.2. Subgroup Analysis of Clinically N0-Stage

Of the 102 patients, in 56 (55%), no suspicious lymph nodes were palpable, and these were categorized as cN0. In these patients, USgFNAC was performed in 99 lymph nodes (Table 1). Cytological results were insufficient for 4 out of 99 (4%) nodes; these nodes were excluded. Of the remaining 95 nodes, 17 (18%) were cytologically malignant.

#### 3.2.1. Short Axis Diameter

The mean short axis diameter was 7.4 mm (SD 3.1) for all aspirated nodes, and 6.6 mm (SD 2.1) and 10.8 mm (SD 4.7) for cytologically confirmed benign and malignant nodes, respectively.

Cytologically confirmed malignant nodes had a significantly larger short axis diameter than cytologically confirmed benign nodes (*p*-value <0.0001).

The short axis diameter of cytologically confirmed malignant nodes was not significantly different between patients with cN0 and cN+ stage (*p*-value = 0.129).

Predicting cytological malignancy for nodes with short axis diameter ≥ 5.5 mm had a sensitivity of 0.94 (95% CI 0.57–1.00), a specificity of 0.26 (95% CI 0.15–0.55), a PPV of 0.22 (95% CI 0.11–0.38), and an NPV of 0.95 (0.59–1.00; Table 2). With a threshold of 4.0 mm (based on the literature) the sensitivity was 1.00 (95% CI 0.80–1.00), the specificity was 0.05 (95% CI 0.01–0.11), the PPV was 0.19 (95% CI 0.09–0.32), and the NPV was 1.00 (95% CI 0.40–1.00; Table 2 and Table 3).

#### 3.2.2. Resistive Index

RI was successfully obtained for 88/95 (92%) of aspirated lymph nodes. Predicting cytological malignancy for nodes with RI ≥ 0.615 had a sensitivity of 1.00 (95% CI 0.77–1.00), a specificity of 0.25 (95% CI 0.15–0.36), a PPV of 0.20 (95% CI 0.06–0.31), and an NPV of 1.00 (0.81–1.00; Table 2).

#### 3.2.3. S/L Ratio

The mean S/L ratio was 0.6 (SD 0.17) for all nodes, and was 0.5 (SD 0.16) and 0.7 (SD 0.16) for cytologically confirmed benign and malignant nodes, respectively. Malignant nodes had a significantly larger S/L ratio than benign nodes (*p*-value <.001). Using S/L ratio to predict cytological malignancy for nodes with a ratio > 0.5 had a sensitivity of 0.88 (95% CI 0.71–1.00), a specificity of 0.46 (95% CI 0.36–0.56), a PPV of 0.26 (95% CI 0.14–0.42), and an NPV of 0.95 (95% 0.82–1.00; Table 2).

#### 3.2.4. Peripheral Vascularization by MFI

Peripheral vascularization obtained by MFI was present in 32/95 (33.7%) nodes. Predicting cytological malignancy had a sensitivity of 0.94 (95% CI 0.56–1.00), a specificity of 0.79 (95% CI 0.70–0.88), a PPV of 0.50 (95% CI 0.27–0.71); and an NPV of 0.98 (0.92–1.00; Table 2 and Table 3).

#### 3.2.5. Absent Hilum Sign

Fatty hilum sign was absent in 28/95 (29.5%) nodes. Predicting cytological malignancy had a sensitivity of 0.82 (95% CI 0.60–1.00), a specificity of 0.82 (95% CI 0.73–0.89), a PPV of 0.50 (95% CI 0.24–0.72), and an NPV of 0.96 (0.89 -0.99; Table 2 and Table 3).

Among nodes with absent hilum sign, peripheral vascularization obtained by MFI had a sensitivity of 0.93 (95% CI 0.50–1.00), a specificity of 0.64 (95% CI 0.36–0.88), a PPV of 0.72 (95% CI 0.40–0.92), and an NPV of 0.90 (0.55–1.00) for the prediction of cytological malignancy (Table 2 and Table 3).

### 3.3. Subgroup Nodes with Short Axis Diameter ≤ 6 mm

Short axis diameter was ≤ 6 mm for 60/203 (29.6%) nodes. 

#### 3.3.1. Resistive Index

RI was successfully obtained for 56/60 (93%) nodes. Predicting cytological malignancy for nodes with RI ≥ 0.615 had a sensitivity of 0.80 (95% CI 0.38–1.00), a specificity of 0.26 (95% CI 0.00–0.58), a PPV of 0.32 (95% CI 0.07–0.30), and an NPV of 86 (0.57–0.98).

#### 3.3.2. S/L Ratio 

Using the S/L ratio to predict cytological malignancy for nodes with a ratio > 0.5 had a sensitivity of 0.82 (95% CI 0.40–1.00), a specificity of 0.61 (95% CI 0.49–0.73), a PPV of 0.32 (95% CI 0.16–0.52), and an NPV of 0.94 (95% 0.79–1.00; Table 2).

#### 3.3.3. Peripheral Vascularization by MFI

Peripheral vascularization obtained by MFI was present in 13/60 (21.7%) nodes. Predicting cytological malignancy had a sensitivity of 0.73 (95% CI 0.33–0.93), a specificity of 0.90 (95% CI 0.79–0.96), a PPV of 0.62 (95% CI 0.30–0.86), and an NPV of 0.94 (0.82–0.98; Table 2 and Table 3).

#### 3.3.4. Absent Hilum Sign

Fatty hilum sign was absent in 20/60 (33.3%) nodes. Predicting cytological malignancy had a sensitivity of 0.91 (95% CI 0.00–1.00), a specificity of 0.80 (95% CI 0.67–0.89), a PPV of 0.50 (95% CI 0.23–0.72), and an NPV of 0.98 (0.86–1.00; Table 2 and Table 3)

## 4. Discussion

Ultrasound enables better assessment of the morphology of small nodes than other modalities [22]. USgFNAC is commonly used to detect metastatic spread and is reported to have a sensitivity of 81% [23]. In a systematic review, USgFNAC has been shown to be much less sensitive for patients with cN0 neck with a pooled sensitivity of 66% (95% CI 54–77%) [24].

Nodal size is an important feature used for selecting nodes for USgFNAC. Van den Brekel et al. showed that different radiologists obtain varying sensitivities, mainly based on selection of lymph nodes being aspirated. The more rigorous the aspiration policy, the higher the sensitivity [20]. In general, it has been concluded by Borgemeester et al. that, apart from features such as round shape, cortical widening, and absence of a hilum, in cN0 necks, nodes should be aspirated when they have a short axis diameter of at least 5–6 mm for level II and 4–5 mm for the rest of the neck levels [25].

Using these small cut-off values, we will have to deal with more reactive lymph nodes as well as more non-diagnostic aspirates. On the other hand, using a larger cut-off diameter for selection will lead to more false negatives. We should also realize that micro metastases and metastases smaller than 4mm will rarely be detected by USgFNAC and these metastases might well be the only metastases present in up to 25% of cN0 necks with clinically occult metastases [26].

Although selection of the nodes to aspirate is important for increasing sensitivity, on the other hand, aspiration can be obviated in lymph nodes that have morphological criteria for malignancy that cannot be ignored in treatment selection. In fact, this means that in lymph nodes that are truly enlarged, necrotic, or otherwise almost certainly malignant, cytological confirmation is not necessary in case of a known primary cancer.

We found that a large, short axis diameter was very reliable in predicting cytological malignancy. In fact, all of the aspirates of lymph nodes with a short axis length of at least 14 mm were tumor positive. Of those with a shorter short axis, 63% were benign.

However, to achieve a high sensitivity, smaller lymph nodes should also be aspirated. Comparing diameter as a criterion with MFI, we found that the short axis criterion with the same sensitivity as peripheral vascularization obtained by MFI yielded a substantially lower specificity (45% vs. 84% in all nodes and 26% vs. 79% in nodes from patients with cN0 neck).

Another important predictor for cytologically confirmed malignancy is the nodal shape, as malignant nodes tend to be more round with a S/L ratio above 0.5 [10,27]. In our study we also found a significantly larger S/L ratio in cytologically malignant nodes than in benign nodes. A ratio >0.5 predicted cytological malignancy correctly in 59% of all nodes, with a sensitivity of 88% and a specificity of 45%. This performance is very similar to that of the short axis diameter with our determined threshold of 6.5 mm. Similar results were obtained in the subset of patients with cN0 neck.

Size and S/L ratio are important features to select nodes for FNAC, but this study shows that selection criteria can be improved when combining them with morphological criteria.

In our study, we evaluated the absence of a fatty hilum sign as the presence of an echogenic hilum in a lymph node can be a sign of a benign lymph node [13]. Including the entire cN0 and cN+ patient group, 82% of the nodes with an absent fatty hilum sign were malignant at cytology, while this was 50% in N0 necks. The sensitivity of this criterion for all lymph nodes and for the lymph nodes in the cN0 necks was 91% and 82%, whereas specificity was 80% and 82%, respectively.

Ghafoori et al. showed that vascular patterns had better performance than size and RI when predicting cytological malignancy of a node in a study of large palpable cervical lymph nodes (accuracy 89%, sensitivity 85%, specificity 93%) [28]. However, in this study only the largest palpable lymph nodes with a mean short axis diameter of 22.6 mm for malignant nodes and 16.6 mm for benign nodes were evaluated, which are large compared with our study. Visualization of morphological changes and vascular patterns is much more difficult in small lymph nodes. MFI is designed to improve the visualization of blood flow, especially in micro vessels [29]. Using MFI, we were able to detect peripheral micro vascularization in small nodes. Peripheral vascularization had a PPV of 50% in nodes from cN0 patients (NPV 98%, sensitivity 94%, specificity 79%), while the PPV was 83% in nodes from all cN stages (NPV 88%, sensitivity 87%, specificity 84%).

In nodes with absent hilum sign and present peripheral vascularization from patients with all cN stages, 94% of the nodes were malignant at USgFNAC, while 72% were malignant for patients with cN0 neck. The sensitivity in both groups is comparable (92% for all patients, 93% for patients with cN0 neck) and specificity is reasonably high (79% and 64%).

The sensitivity of USgFNAC in patients with cN0 is reported to be in the range of 42–73% [30]. The specificity of USgFNAC is always in the order of 100% as false positive cytology is rare. The difference in sensitivity is mainly attributable to selection of the lymph nodes to aspirate and for aspiration technique. Selection of the most suspicious lymph nodes is on the one hand guided by location of the primary tumor, with known patterns of metastases, and on the other hand by size, shape and morphological criteria. In our study we found clear evidence that selection of the lymph nodes for aspiration can be improved by using not only size and shape, but also peripheral vascularization as detected by MFI. In nodes with a short axis diameter of 6 mm and smaller, 62% of the nodes with present peripheral vascularization and 50% with absent fatty hilum sign were malignant. In those small nodes, absence of fatty hilum sign had a higher sensitivity (91%) than peripheral vascularization (73%), but a lower specificity (80% vs. 90%). The positive predictive value was highest when combining absent fatty hilum sign and peripheral vascularization, although only a few nodes showed this combination. Assessment of peripheral vascularization with MFI can be done while adding hardly any examination time.

However, not all metastatic lymph nodes have peripheral vascularization or an absent hilum, so absence of these features should not be used as the sole reason not to aspirate from these lymph nodes. The size and location in the neck, relative to the primary tumor, are important selection criteria as well.

Adding RI measurements is time consuming, especially in tiny nodes. In large necrotic nodes, the RI is sometimes not measurable. In accordance with the findings of Ahuja et al., our results show that the intravascular pattern appears more useful in distinguishing malignant from benign nodes than the RI [31].

Because we tested these criteria in patients treated with organ preservation, we only have cytological results and no histopathology of the neck dissection. In general, USgFNAC overlooks 20–40% of the neck sides with occult metastases, mostly very small nodes [4]. Some of these micro metastases likely will not have features related to size, shape, hilum, or vascularization. As a consequence, US criteria for these small metastases are likely never to be found and a certain limit of the accuracy has to be accepted. However, our study reflects the clinical workflow in most hospitals, where USgFNAC is used together with PET-CT (or other modalities) for the purpose of nodal staging and treatment selection. The results of our study can therefore be used to better identify nodes for which USgFNAC should be performed.

Another issue is that in some patients with a known head and neck cancer and already clinically apparent lymph node metastases, nodes with US features (large diameter, peripheral vascularization, no hilum) that are almost pathognomonic for metastases are found on ultrasound. For these patients, cytological proof has no clinical significance, as these nodes need treatment, and a negative cytology is not trustworthy. From our study, we can conclude that lymph nodes with a minimal axial diameter larger than 14 mm, but also lymph nodes without a hilum and with peripheral vascularization, have such a high incidence of positive cytology that one could consider refraining from aspiration in these nodes and categorize them as malignant, based on morphological criteria.

## 5. Conclusions

Detection of peripheral vascularization in lymph nodes using MFI has, similar to the loss of fatty hilum, a high predictive value in predicting metastases by USgFNAC. Peripheral vascularization has a high sensitivity and can also be (quickly) assessed in small nodes, such as nodes from cN0 necks. Although in all necks peripheral vascularization has a similar PPV as absent fatty hilum, in nodes with clinical N0-stage the sensitivity is remarkably higher (94%) than for the absent fatty hilum sign (82%). Peripheral vascularization should be used in combination with an absent fatty hilum sign, nodal size and shape to select lymph nodes for USgFNAC. As USgFNAC can also have false negative cytological results, a negative cytology in nodes which show these US criteria should be distrusted and USgFNAC should be repeated.

## Figures and Tables

**Table 1:** Patient date.

	All cN Stages			cN0-Stage		
	Total	Female	Male	Total	Female	Male
N patients	102	27 (26%)	73 (72%)	56	16 (29%)	38 (68%)
Mean age (range)	65 (34–87)	63 (45–87)	65 (34–84)	65 (34–87)	63 (51–87)	66 (34–84)
N aspirated nodes	211			99		
Mean nodes/patient (range)	2.07 (1–5)			1.77 (1–5)		

**Table 2:** Predictive performance of features in different subsets of nodes.

Data	Specificity	Sensitivity	NPV	PPV	Threshold
**all nodes**					
*p*. vascularization	0.84 (0.77–0.90)	0.87 (0.80–0.93)	0.88 (0.80–0.94)	0.83 (0.74–0.90)	
absent fatty hilum	0.84 (0.77–0.90)	0.84 (0.77–0.90)	0.86 (0.76–0.92)	0.82 (0.72–0.90)	
short axis diameter	0.45 (0.19–0.81)	0.88 (0.80–0.95)	0.82 (0.59–0.89)	0.59 (0.45–0.82)	6.5 ^1^
short axis diameter	0.25 (0.17–0.35)	0.95 (0.89–0.98)	0.84 (0.68–0.94)	0.53 (0.43–0.62)	6.0 ^2^
resistive index	0.54 (0.34–0.70)	0.88 (0.78–0.93)	0.85 (0.72–0.92)	0.61 (0.50–0.74)	0.705 ^1^
S/L ratio ^3^	0.45 (0.37–0.53)	0.88 (0.82–0.93)	0.82 (0.69–0.90)	0.59 (0.49–0.67)	0.5
**cN0 patients**					
*p*. vascularization	0.79 (0.70–0.88)	0.94 (0.56–1.00)	0.98 (0.92–1.00)	0.50 (0.27–0.71)	
asent fatty hilum	0.82 (0.73–0.89)	0.82 (0.60–1.00)	0.96 (0.89–0.99)	0.50 (0.24–0.72)	
short axis diameter	0.26 (0.15–0.55)	0.94 (0.57–1.00)	0.95 (0.59–1.00)	0.22 (0.11–0.38)	5.5 ^1^
short axis diameter	0.05 (0.01–0.11)	1.00 (0.80–1.00) *	1.00 (0.40–1.00) *	0.19 (0.09–0.32)	4.0 ^2^
restistive index	0.25 (0.15–0.36) *	1.00 (0.77–1.00) *	1.00 (0.81–1.00) *	0.20 (0.06–0.31)	0.615 ^1^
S/L ratio ^3^	0.46 (0.36–0.56)	0.88 (0.71–1.00)	0.95 (0.82–1.00)	0.26 (0.14–0.42)	0.5
**absent fatty hilum**					
*p*. vascularization	0.71 (0.45–0.89)	0.92 (0.85–0.97)	0.67 (0.38–0.85)	0.94 (0.86–0.98)	
**cN0 and absent fatty hilum absent**					
*p*. vascularization	0.64 (0.36–0.88)	0.93 (0.50–1.00)	0.90 (0.55–1.00) *	0.72 (0.40–0.92)	
**short axis diameter** **mm ≤ 6**					
*p*. vascularization	0.90 (0.79–0.96)	0.73 (0.33–0.93)	0.94 (0.82–0.98)	0.62 (0.30–0.86)	
absent fatty hilum	0.80 (0.67–0.89)	0.91 (0.00–1.00)	0.98 (0.86–1.00)	0.50 (0.23–0.72)	
resistive index	0.26 (0.00–0.58)	0.80 (0.38–1.00)	0.86 (0.57–0.98) *	0.19 (0.07–0.30)	0.615 ^1^
S/L ratio ^3^	0.61 (0.49–0.73)	0.82 (0.40–1.00)	0.94 (0.79–1.00)	0.32 (0.16–0.52)	0.5

The given confidence intervals are 95% bias-corrected accelerated bootstrap confidence intervals when possible. * 95% binomial proportion confidence interval. ^1^ threshold determined such that sensitivity ≥ sensitivity for peripheral vascularization. ^2^ threshold based on the literature. ^3^ ratio short axis diameter / long axis diameter.

**Table 3:** Numbers of cytologically proven malignant and benign nodes by categories of sonographic features.

	Nodes at All cN Stages					Nodes at cN0 Stages					Nodes with Short Axis mm ≤ 6 mm				
Features	N	Mal ^3^	*%.*	Ben ^4^	*%.*	N	Mal ^3^	*%.*	Ben ^4^	*%*	N	Mal ^3^	*%*	Ben ^4^	*%*
hilus + ^1^	106	15	14%	91	86%	67	3	4%	64	96%	40	1	3%	39	98%
hilus − ^1^	97	80	82%	17	18%	28	14	50%	14	50%	20	10	50%	10	50%
*p*. vasc + ^2^	100	83	83%	17	17%	32	16	50%	16	50%	13	8	62%	5	38%
*p*. vasc − ^2^	103	12	12%	91	88%	63	1	2%	62	98%	47	3	6%	44	94%
hilus−/ *p*. vasc +	79	74	94%	5	6%	18	13	72%	5	28%	9	7	78%	2	22%
hilus−/ *p*. vasc -	18	6	33%	12	67%	10	1	10%	9	90%	11	3	27%	8	73%

^1^ Hilus +/−: present and absent fatty hilum sign. ^2^ *p*. vasc +/−: present and absent peripheral vascularization. ^3^ mal = cytologically proven malignant nodes. ^4^ ben= cytologically proven benign nodes.

## Data Availability

The data used to support the findings of this study are available from the corresponding author upon request.
